# Linearization of genome sequence graphs revisited

**DOI:** 10.1016/j.isci.2021.102755

**Published:** 2021-06-19

**Authors:** Anna Lisiecka, Norbert Dojer

**Affiliations:** 1 Institute of Informatics, University of Warsaw, Banacha 2, 02-097 Warsaw, Poland

**Keywords:** Bioinformatics, Computer science, Genomics

## Abstract

The need to include the genetic variation within a population into a reference genome led to the concept of a genome sequence graph. Nodes of such a graph are labeled with DNA sequences occurring in represented genomes. Due to double-stranded nature of DNA, each node may be oriented in one of two possible ways, resulting in marking one end of the labeling sequence as in-side and the other as out-side. Edges join pairs of sides and reflect adjacency between node sequences in genomes constituting the graph. Linearization of a sequence graph aims at orienting and ordering graph nodes in a way that makes it more efficient for visualization and further analysis, e.g. access and traversal. We propose a new linearization algorithm, called ALIBI – Algorithm for Linearization by Incremental graph BuIlding. The evaluation shows that ALIBI is computationally very efficient and generates high-quality results.

## Highlights

• We propose ALIBI – a new algorithm for linearization of genome sequence graphs
• ALIBI yields less feedback arcs and reversing joins than competing methods
• ALIBI shows high efficiency and scales well to large graphs

## Graphical abstract

**Figure:**
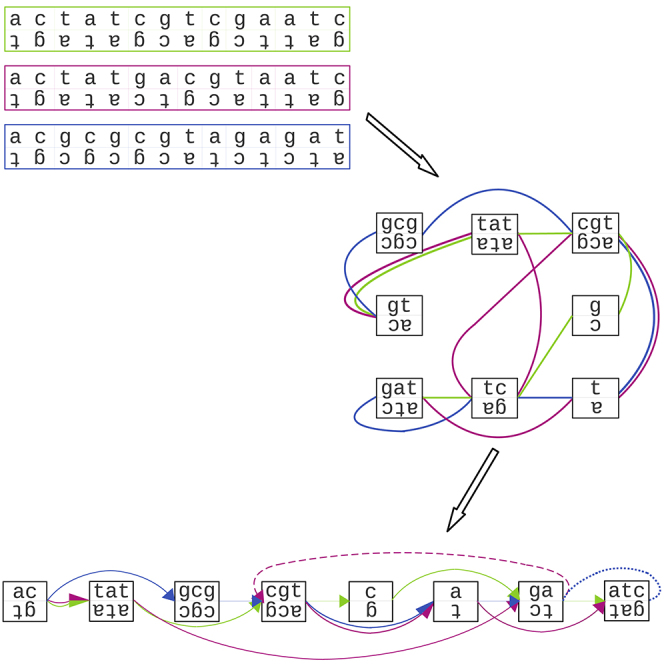

## Introduction

Reference genomes serve as most important genetic resources for particular populations. They provide coordinate systems for gene annotations, targets for sequencing read mapping and downstream analysis, including variant detection, open chromatin areas and protein binding sites identification, 3-dimensional structure reconstruction, etc. Availability of thousands of individual genomes per species revealed some imperfections of this concept. For example, there are genetic variants that cannot be easily described with respect to the reference genome Horton et al. (2008). Moreover, when used as a target for read mapping, it introduces bias toward the reference alleles Brandt et al. (2015). To overcome these drawbacks, the idea of common representation of a variety of genomes within a population has evolved, leading to the concept of *genome sequence graph*.

**Figure 1:**
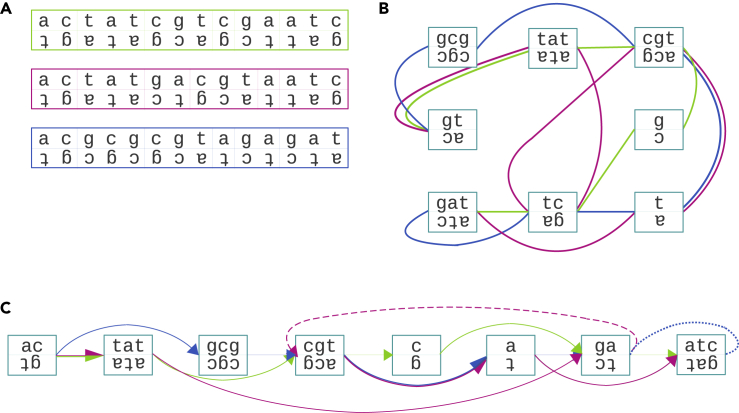
From DNA sequences to linearized sequence graph (A) Input DNA sequences ACGCGCGTAGAGAT, ACTATCGTCGAATC and ACTATGACGTAATC (each depicted with its reverse complement), divided into fragments aligned with fragments of other sequences. (B) Sequence graph: nodes represent blocks of aligned DNA fragments, edges join nodes labeled with fragments adjacent in sequences. Paths constituted by green/purple/blue edges represent respective input sequences. For visibility, pairs of nodes labeled with DNA fragments adjacent in multiple sequences are represented by separate edges rather than edge weights. (C) The same graph after linearization. Nodes are ordered (from left to right) and oriented – the choice of the primary strand results in denominating left sides as in-sides and right sides as out-sides. Consequently, edges are classified as either forward arcs (solid), feedback arcs (dashed) or reversing joins (dotted). The presented linearization minimizes the number of edges of the last two types.

Genome sequence graphs are bidirected graphs enhanced with additional structure that allows to represent the relationship within a set of similar genomic sequences (see Figures 1A and 1B). Each node of a sequence graph is labeled with DNA sequence. Each edge is attached to either left or right side of each incident node, representing the 5′ or 3′ end of the sequence, respectively. A directed path *orients* each visited node in one of two possible ways, corresponding to two strands of the DNA fragment labeling the node. Concatenating strand sequences from oriented consecutive nodes on a path yields sequence *represented by* this path. A sequence graph with a collection of its directed paths covering all graph edges represents the set of sequences determined by these paths. Additionally, each edge is assigned a *weight* equal to the number of times it is traversed by paths corresponding to represented genomes.

Genome sequence graphs are an intuitive way to represent the genetic variation, in particular large-scale structural variants (e.g. insertions, deletions, and translocations) in a collection of sufficiently diverse set of genomes. They are applied in several fields, including pangenome modeling (The Computational Pan-Genomics Consortium 2018; Dziadkiewicz and Dojer 2020), improving the quality of read mapping and variant calling (Novak et al., 2017), whole-genome alignment construction (Kehr et al., 2014), or haplotype determination (Paten et al., 2017).

Linearization of a sequence graph aims at reasonable ordering and orienting nodes (see Figure 1C). The result may influence its usability in several aspects, including:

• visual analysis,
• convenience and interpretability of introduced common coordinate system identifying genetic loci of all underlying genomes,
• efficiency of graph-based analysis: searching, simulation, genome comparison etc.

Generally, the more consistent the underlying genomes are with the orientation and ordering of graph nodes, the easier to use the graph is. Ideally, the paths representing genomes should be made up of only *forward arcs*, i.e. edges joining out-sides with in-sides such that the outside node precedes the inside node. Therefore, the quality of linearization may be quantified using the following metrics (see Figure 1C):

• *Weighted feedback arc* (WFA) – the sum of weights of all *feedback arcs*, i.e. backward pointing edges
• *Weighted reversing join* (WRJ) – the sum of weights of all *reversing joins*, i.e. edges joining two in- or two out-sides.

**Figure 2:**
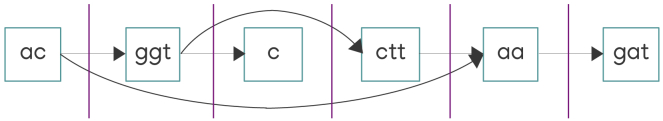
Average cut width (ACW) Purple vertical lines represent the cuts imposed by the linearization of a sequence graph. Consecutive cuts have width 2, 3, 2, 2, and 1 and the average cut width of this graph is 2.

Haussler et al. (2018) proposed an additional metric, called *average cut width* (ACW), i.e. mean number of edges crossing graph cuts placed between any two consecutive nodes (see Figure 2).

A two-step approach to the linearization problem was proposed in the study by Haussler et al. (2018). In the first step, sequence graph is transformed to a directed graph by orienting nodes and ignoring edges joining two in- or two out-sides. In the second step, the nodes of the graph are ordered using either one of well-known heuristics for the feedback arc set problem or an algorithm proposed by authors.

In the current paper, we develop research in this area. We propose a new approach to the linearization problem, in which graph nodes are oriented and ordered jointly. Our algorithm is comprehensively evaluated and compared to previous approaches.

## Results

### Overview of Algorithm for Linearization by Incremental graph BuIlding

Tha main idea of our algorithm is to build the linearized graph gradually by adding edges in a decreasing order of weights. We try to establish as many as possible heavy forward arcs and this way reduce both WFA and WRJ. The algorithm starts from the empty graph with arbitrarily oriented nodes and trivially ordered singleton connected components. When a new edge joins two components, their orders are merged. Therefore, at each step of the algorithm, every connected component is separately linearized and, consequently, every edge is classified as reversing join, feedback arc, or forward arc. The pseudocode of Algorithm for Linearization by Incremental graph BuIlding (ALIBI) is given in Algorithm 1.

Algorithm 1 **Algorithm for Linearization by Incremental graph BuIlding**

1. **input** genome sequence graph (V,E)
2. **output** linearized graph $\left(V^{\prime },E^{\prime }\right)$
3. Set arbitrary orientation on each v∈V
4. $\begin{array}{cccccccccc} \left(V^{\prime },E^{\prime }\right)\leftarrow \left(V,Ø\right) & & & & & & & & & \;▹\;\mathrm{initialize}\;\mathrm{linearized}\;\mathrm{graph} \end{array}$
5. Sort *E* in descending order of weights
6. **for** e∈E **do**
  7. **if** *e* joins nodes belonging to the same connected component **then**
    8. **if** *e* joins two in- or two out-sides **then**
      9. Add *e* to $E^{\prime }$ as reversing join
    10. $\begin{array}{cccccccccc} \mathrm{else} & & & & & & & & & \;▹\;e\;joins\;one\;in-\;and\;one\;out-side \end{array}$
      11. **if** the out-side node precedes the in-side node **then**
        12. Add *e* to $E^{\prime }$ as forward arc
      13. **else if** there is a forward arc path from in-side node to out-side node **then**
        14. Add *e* to $E^{\prime }$ as feedback arc
      15. **else**
        16. Reorder the component such that the out-side node precedes the in-side node
        17. Add *e* to $E^{\prime }$ as forward arc
  18. $\begin{array}{cccccccccc} \mathrm{else} & & & & & & & & & \;▹\;e\;joins\;nodes\;belonging\;to\;two\;different\;connected\;components \end{array}$
    19. **if** *e* joins two in- or two out-sides **then**
      20. Reverse one of the connected components bridged by *e*
    21. $\begin{array}{llll} Merge\;component\;orders & & & \;▹\;out-side\;node\;component\;precedes\;in-side\;node\;component \end{array}$
    22. Add edge *e* to $E^{\prime }$ as forward arc
23. **end for**

The justification of our approach, as well as implementation details and complexity analysis, is given in the Methods section. See also Figure 6, Figure 7, Figure 8.

### Evaluated approaches

We compared ALIBI against two linearization algorithms implemented in vg tool (Garrison et al., 2018). Both vg methods follow the two-step approach proposed by Haussler et al. (2018) and share the algorithm that performs the node orienting step. The node ordering step in these methods are different: Eades is the implementation of a well-known heuristic for the feedback arc set problem of Eades et al. (1993) and FP is the flow procedure proposed in (Haussler et al., 2018) – this algorithm focuses on minimizing average cut width.

### Performance on simulated data

We prepared simulated data following the procedure of Haussler et al. (2018). Namely, we took a 37287bp-long fragment of human genome and applied to it a series of structural variations using the Bioconductor package RSVSim (Bartenhagen and Dugas 2013). Introduced variations included deletions, insertions, inversions, and duplications of lengths 20, 20, 200, and 500, respectively. Each simulation generated the same number of variations of each type, varying from 5 to 11. For each of these numbers, 10 rearranged genomes were created and passed to the msga command of the vg, which generated a sequence graph in .gfa format.

**Figure 3:**
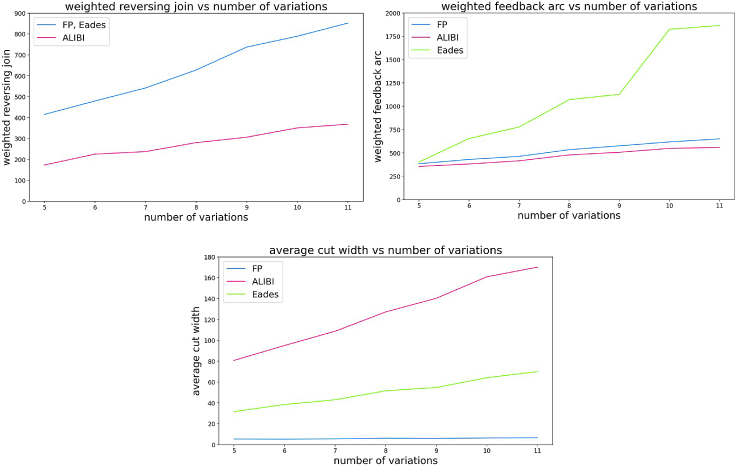
Quality metrics on simulated data: WRJ (top left), WFA (top right) and ACW (bottom)

Figure 3 presents the WRJ, WFA, and ACW results of the algorithms on the simulated data sets. The total weight of reversing joins in ALIBI is approximately half the size of FP and Eades algorithms (both have identical WRJ because they share the algorithm that performs the node orienting step). In terms of feedback arcs, ALIBI is slightly better than FP, while Eades algorithm is surprisingly the weakest. As expected, FP outperforms all other methods in terms of ACW.

### Performance on *Escherichia coli* genomes

We also prepared two series of graphs build from real genomes of *Escherichia coli* K-12 strain. In the first series, we took 4 genomes and created graphs using vg msga with the parameter -m restricting the length of sequence labeling nodes set to 32, 64, 128, 256, and 512. In this way, we obtained a series of graphs with similar complexity but varying numbers of nodes and edges. In the second series we build graphs with default parameters of vg msga and the number of *E. coli* genomes varying from 10 to 150 (see Table S1 for the full list of genomes).

**Figure 4:**
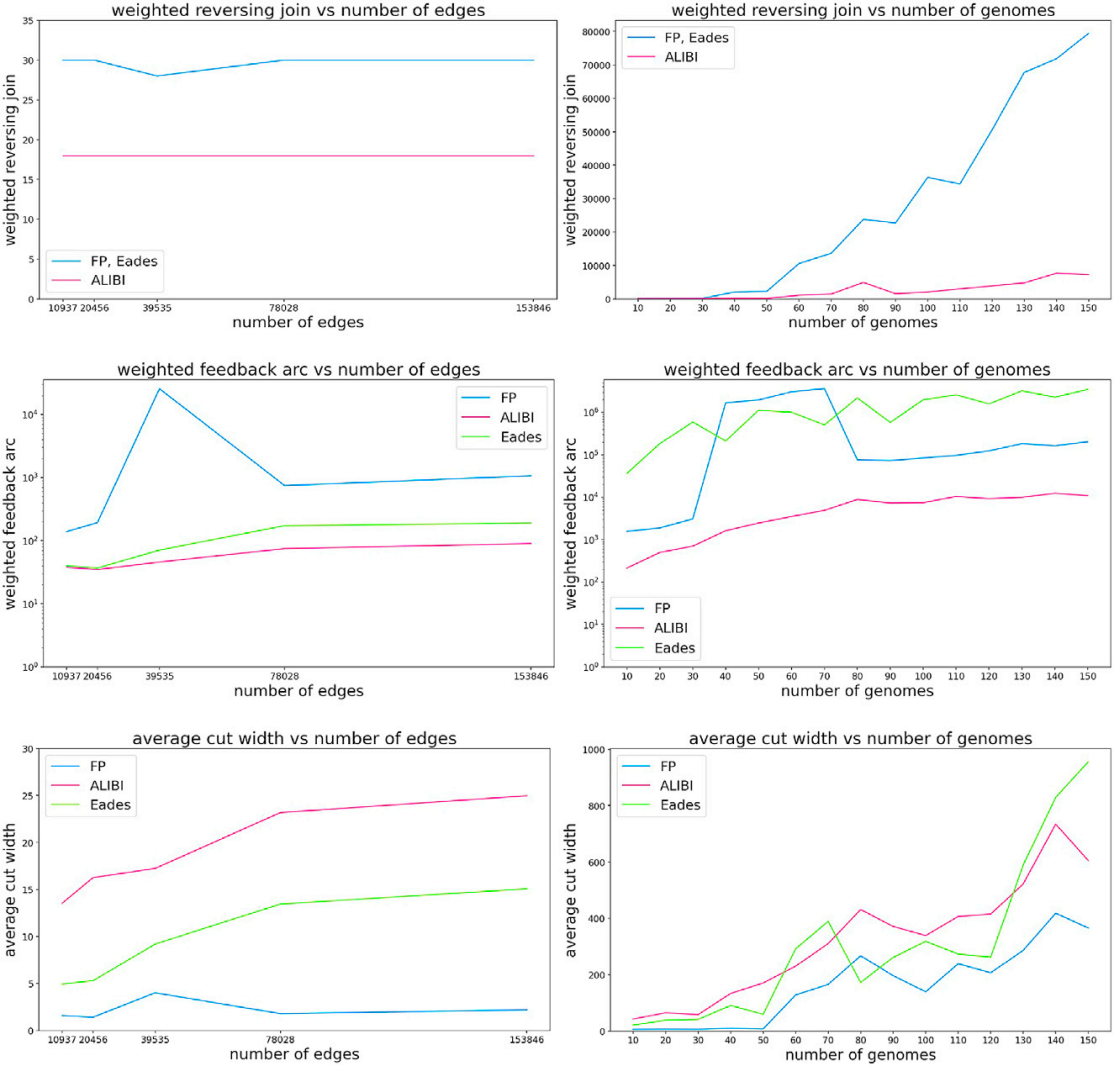
Quality metrics on *E. coli* genomes: WRJ (top), WFA (middle) and ACW (bottom) Plots present dependence on the size of the graph (left, all graphs are constructed from 4 genomes, GenBank: AP009048, AP012306, CP000948, U00096) and on the number of genomes (right, see Table S1 for the list of genomes).

Figure 4 presents results obtained on *Escherichia coli* data sets. Similarly to simulated data results, FP has the lowest ACW, while ALIBI outperforms the competitors in both WRJ and WFA. However, the differences in WFA are surprisingly extreme here – in some cases, Eades or FP algorithms have WFA over 500 times larger than ALIBI (note the logarithmic scale on the Y axis). Moreover, the relationship between the metrics and the number of genomes is not clear, probably due to highly varying complexity of genome graphs.

**Figure 5:**
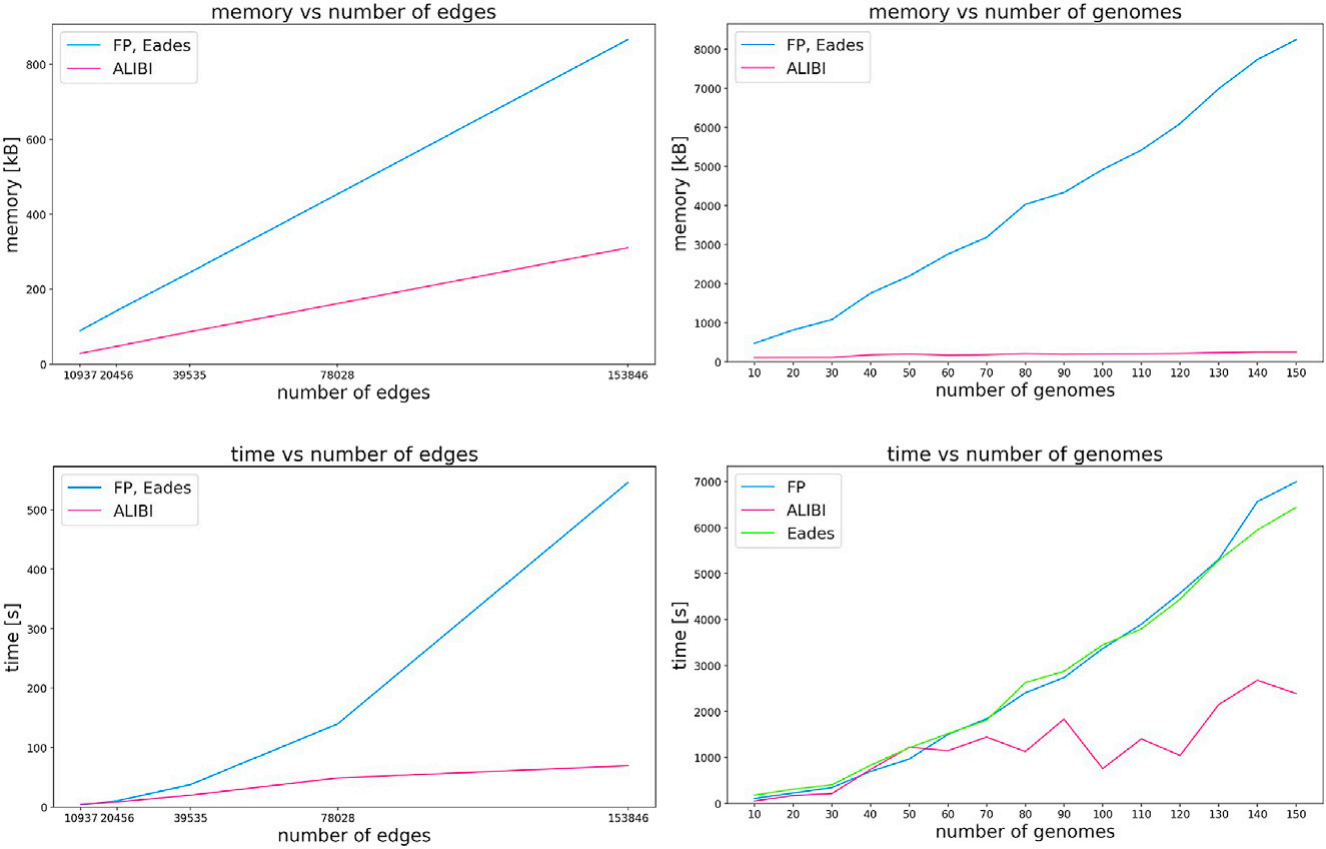
Efficiency of algorithms on *E. coli* genomes: occupied memory (top) and computation time (bottom) Plots present dependence on the size of the graph (left, all graphs are constructed from 4 genomes, GenBank: AP009048, AP012306, CP000948, U00096) and on the number of genomes (right, see Table S1 for the list of genomes).

Figure 5 summarizes computational efficiency of the algorithms. ALIBI performs best in both experimental settings and with respect to both time and memory resources. All algorithms scale roughly linearly with respect to both number of edges and number of genomes. However, some irregularities are visible in the plots presenting the dependence with respect to the number of genomes, which suggests that the graph complexity significantly affects the computation cost.

**Figure 6:**
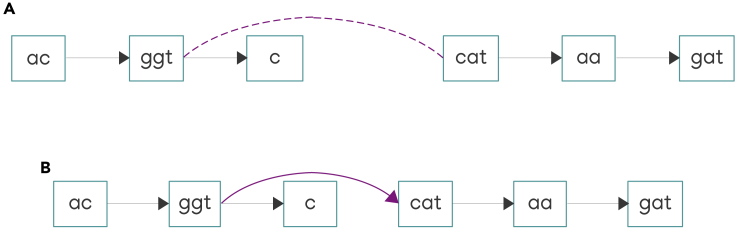
Adding edges joining an out-side with an in-side of nodes from different connected components (A) The purple edge joins the out-side of the node labeled with “ggt” sequence and the in-side of the node labeled with “cat” sequence. (B) The edge is added to the graph without any change in node orientations. The nodes from the left component precede the nodes from the right component.

**Figure 7:**
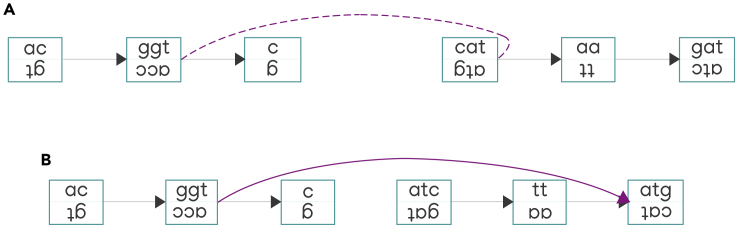
Adding edges joining out-sides of nodes from different connected components (A) The purple edge joins the out-side of the node labeled with “ggt/acc” sequence and the out-side of the node labeled with “cat/atg” sequence. (B) After changing the order and orientations of the nodes from the right-hand side connected component (i.e. in-side becomes out-side, out-side becomes in-side, and node sequence changes to its reverse complement), the purple edge joins the out-side of the node labeled with “ggt/acc” sequence and the in-side of the node labeled with “atg/cat” sequence. The purple edge can now be added to the graph.

**Figure 8:**
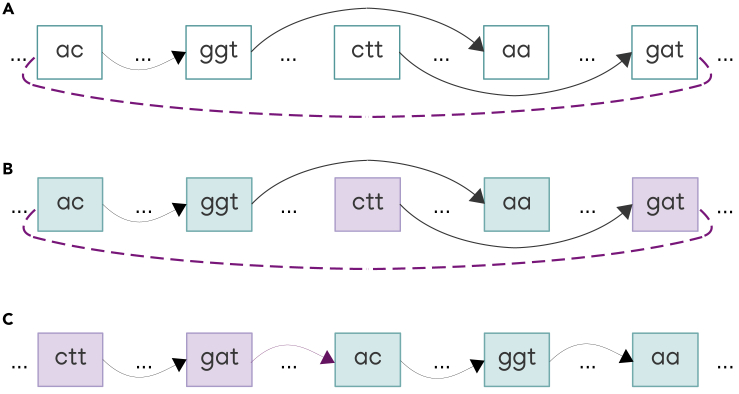
Adding an edge inconsistent with the order on a connected component using Pearce-Kelly algorithm (A) The purple edge connects the in-side of the node labeled with “ac” sequence and the out-side of the node labeled with “gat” sequence. There is no path from node “ac” to node “gat.” Reordering will affect the region between these nodes. (B) Identification of the nodes from the affected region that are either reachable from node “ac” (light blue nodes) or from which node “gat” is reachable (light purple nodes). The gaps denoted by dots may contain other nodes. (C) Light purple and light blue nodes are permuted such that all the light purple nodes precede all the light blue nodes and the original order within light purple nodes is preserved, as well as within light blue nodes. Positions of all other nodes remain unaffected.

## Discussion

In the current paper, we proposed ALIBI, a novel linearization algorithm that jointly orients and orders graph nodes. We compared our method with two state of the art algorithms presented in the study by Haussler et al. (2018).

The evaluation shows that ALIBI substantially outperforms its competitors in two out of three quality metrics: weighted reversing join and weighted feedback arc, while flow procedure of the study by Haussler et al. (2018) achieves best results in terms of average cut widths. Moreover, ALIBI is the fastest and has the lowest memory requirements.

### Limitations of the study

The required computational resources of ALIBI and other linearization algorithms depend on the structure of the input graph rather than on the number and size of the underlying genome sequences. Consequently, the computational cost of linearization of graphs build from the same genome data set using different tools may substantially differ.

## STAR★methods

### Key resources table

**Table:** 

REAGENT or RESOURCE	SOURCE	IDENTIFIER
**Software and algorithms**		

vg v1.10.0	Garrison et al. (2018)	https://github.com/vgteam/vg
RSVSim 1.20.0	Bartenhagen and Dugas (2013)	http://www.bioconductor.org/packages/release/bioc/html/RSVSim.html
ALIBI	This paper	https://github.com/anialisiecka/alibi

### Resource availability

#### Lead contact

Further information and requests for resources should be directed to and will be fulfilled by the lead contact, Norbert Dojer (dojer@mimuw.edu.pl).

#### Materials availability

This study did not generate new unique reagents.

#### Data and code availability

All original code has been deposited at https://github.com/anialisiecka/alibi and is publicly available. Any additional information required to reanalyze the data reported in this paper is available from the lead contact upon request.

### Method details

#### Bidirected graphs and genome sequence graphs

The notion of *bidirected graph* was introduced by Edmonds and Johnson (1970). In a bidirected graph every node has two *sides*, called *left* and *right* and each edge endpoint is incident with one side of a particular node. Formally, each edge in a bidirected graph (V,E) is a tuple $\left(v,s,v^{\prime },s^{\prime }\right)$, where $v,v^{\prime }\in V$ and $s,s^{\prime }\in \left\{left,right\right\}$ indicate the incident sides of *v* and *v*′, respectively. This is conceptually similar to directed graphs, where edges are incident with in- or out-sides of nodes, but in bidirected graphs the sides of edge endpoints are independent (i.e. edges may have one left and one right side, as well as both left or both right sides). Thus we can consider directed graphs as a special case of bidirected graphs.

A *path* in a bidirected graph *G* is a sequence $e_{1},e_{2},\ldots ,e_{k}$ of edges such for each pair of consecutive edges $e_{i}=\left(v_{i},s_{i},{v_{i}}^{\text{′}},{s_{i}}^{\text{′}}\right)$ and $e_{i+1}=\left(v_{i+1},s_{i+1},{v_{i+1}}^{\text{′}},{s_{i+1}}^{\text{′}}\right)$ we have ${v_{i}}^{\text{′}}=v_{i+1}$. Path is *directed* if additionally ${s_{i}}^{\text{′}}\neq s_{i+1}$, i.e. if each node is exited on the other side it is entered. A *connected component* of a bidirected graph is a maximal set of nodes such that each pair of nodes is connected by a (not necessarily directed) path.

#### Linearization of a bidirected graph

Let *G* = (*V*, *E*) be a bidirected graph and let $V^{\prime }\subseteq V$. A *linearization* of *V*′ is given by a pair of functions (*a*, *ord*), where:

• *nodes orienting function*$a:V^{\prime }\to \left\{-1,1\right\}$ establishes labeling of node sides as in- or out-sides in the following way:
  – if a(v)=1, then left side of *v* is labeled as in-side and right side as out-side,
  – if a(v)=−1, then left side of *v* is labeled as out-side and right side as in-side.
• *nodes ordering function*$ord:V^{\prime }\to \left\{1,\ldots ,\left|V\right|\right\}$ is a bijection establishing the linear order on *V*′ (i.e. node *v* precedes node *v*′ iff *ord*(*v*) < *ord*(*v′*)).

The linearization implies the following classification of edges joining *V*′-nodes (see Figure 2 (left)):

• *reversing joins* are edges incompatible with node orientations, i.e. joining two in-sides or two out-sides,
• *feedback arcs* are compatible with node orientations but incompatible with their order, i.e. they join out-side of a node *v* with in-side of a node *v*′ satisfying $ord\left(v\right)\geq ord\left(v^{\prime }\right)$,
• *forward arcs* are compatible with both orientations and order, i.e. they join out-side of a node *v* with in-side of a node *v*′ satisfying *ord*(*v*) < *ord*(*v′*).

If the linearized graph has no reversing joins, it is actually a directed graph with sorted nodes. If, additionally, it has no feedback arcs, it is topologically sorted directed acyclic graph.

#### Adding edges to the graph

The following two theorems give the conditions, under which a new edge may be classified as forward arc.

**Theorem 1.** Let *G*_1_ and *G*_2_ be two different linearized connected components with sets of nodes *V*_1_ and *V*_2_, respectively. Assume that a new edge e joining a *V*_1_-node with a *V*_2_-node is added to the graph. Then there exists a linearization of *V*_1_∪*V*_2_ in which e is classified as forward arc and the classification of all edges in both *G*_1_ and *G*_2_ does not change.

*Proof.* Let $\left(a_{1},ord_{1}\right)$ and $\left(a_{2},ord_{2}\right)$ be the given linearizations of *V*_1_ and *V*_2_, respectively. We consider two cases:

1. Edge *e* joins one out- and one in-side. Without loss of generality we may assume that *e* joins an out-side of a node *v*_1_∈*V*_1_ with an in-side of a node *v*_2_∈*V*_2_. Then the linearization (*a*, *ord*) of *V*_1_∪*V*_2_ given by formulas

$$a\left(v\right)=\left\{ \begin{array}{cc} a_{1}\left(v\right) & \text{if }v\in V_{1}\\ a_{2}\left(v\right) & \text{if }v\in V_{2} \end{array}\right.\;ord\left(v\right)=\left\{ \begin{array}{cc} ord_{1}\left(v\right) & \text{if }v\in V_{1}\\ \left|\;V_{1}\right|+ord_{2}\left(v\right) & \text{if }v\in V_{2} \end{array}\right.$$

satisfies the requirements of the theorem (see Figure 6).

2. Edge *e* joins two out- or two in-sides. In order to make *e* a forward arc we have to reverse the order and orientation of nodes in either component (note that reversing doesn’t affect the classification of its inner edges). If *e* joins out-sides of *v*_1_∈*V*_1_ and *v*_2_∈*V*_2_, the following linearization (*a*, *ord*) of *V*_1_∪*V*_2_ does the job (see Figure 7):

$$a\left(v\right)=\left\{ \begin{array}{cc} a_{1}\left(v\right) & \text{if }v\in V_{1}\\ -a_{2}\left(v\right) & \text{if }v\in V_{2} \end{array}\right.\;ord\left(v\right)=\left\{ \begin{array}{cc} ord_{1}\left(v\right) & \text{if }v\in V_{1}\\ \left|V_{1}\right|+\left|V_{2}\right|-ord_{2}\left(v\right)+1 & \text{if }v\in V_{2} \end{array}\right.$$

The case with two in-sides can be handled analogously.

Consider a linearized graph *G* = (*V*, *E*) and two nodes $v_{1},v_{2}\in V$. We say that *v*_2_ is *forward-accessible* from *v*_1_ (denoted $v_{1}⇝v_{2}$) iff there is a directed path from *v*_1_ to *v*_2_ consisting of forward arcs only.

**Theorem 2.** Let (*a*_*c*_, *ord*_*c*_) be a linearization on a connected component $G_{c}=\left(V_{c},E_{c}\right)$. Assume that a new edge e joining nodes $v_{1},v_{2}\in V_{c}$ such that $ord_{c}\left(v_{1}\right)\leq ord_{c}\left(v_{2}\right)$ is added to the graph in the way that doesn’t affect the classification of *E*_*c*_-edges. Then:

1. *If e joins two out- or two in-sides, e must be classified as reversing join.*
2. *If e joins out-side of* *v*_2_ *with in-side of* *v*_1_ *and* $v_{1}⇝v_{2}$*, e must be classified as feedback arc.*
3. *Otherwise e may be classified as forward arc.*

*Proof.* We will separately prove the three statements from the theorem.

1. Since *G*_*c*_ is a connected component, reversing orientation of one node in *V*_*c*_ without violating edge classification implies reversing orientation of all the other *V*_*c*_-nodes. Consequently, there is no linearization on *G*_*c*_ that reverses the orientation of only one of the nodes $v_{1},v_{2}$ and saves the classification of all the *E*_*c*_-edges.
2. Appending *e* to the path from the assumption results in a cycle, which cannot consist of forward arcs only. Thus *e* cannot be classified as forward arc without disturbing the classification of the edges on the path from *v*_1_ to *v*_2_.
3. Since the assumption of the statement 1 is not fulfilled, *e* joins one in- and one out-side. Since the assumptions of the statement 2 are not fulfilled, $v_{2}\neq v_{1}$ and, consequently, $ord_{c}\left(v_{1}\right)<ord_{c}\left(v_{2}\right)$. If *e* joins out-side of *v*_1_ with in-side of *v*_2_, linearization (*a*_*c*_, *ord*_*c*_) classifies *e* as forward arc. Otherwise the nodes must be reordered such that *v*_2_ precedes *v*_1_. This can be done using the approach applied by Pearce and Kelly (2007) in their dynamic algorithm for topologically sorting directed acyclic graphs. The method consists of:

• Identifying two sets of nodes:

$$V_{F}=\left\{v\in V_{c}\;|\;v_{1}⇝v\wedge ord_{c}\left(v\right)\leq ord_{c}\left(v_{2}\right)\right\}$$

$$V_{B}=\left\{v\in V_{c}\;|\;v⇝v_{2}\wedge ord_{c}\left(v_{1}\right)\leq ord_{c}\left(v\right)\right\}$$

Note that *V*_*F*_ and *V*_*B*_ are disjoint, because otherwise we would have *v*_1_⇝*v*_2_.

• Updating the positions of nodes in *V*_*F*_∪*V*_*B*_ such that all *V*_*B*_-nodes precede all *V*_*F*_-nodes and the original order within both *V*_*F*_ and *V*_*B*_ is preserved (see Figure 8).

The above theorems justify updating the graph with new edges according to the following rules:

1. Edges joining nodes belonging to different connected components are always classified as forward arcs. There are two cases:
  (a) If the edge joins one in- and one out-side, node orientations remain unchanged. Orders on joined components are merged in the way that all the nodes from the component connected with the edge in an out-side precede all the nodes from the other component (see Figure 6).
  (b) If the edge joins two in- or two out-sides, the order and orientations of nodes from one of the connected components are reversed (see Figure 7). After this step, the orders on both components are merged as in the previous case.
2. Edges joining nodes belonging to the same connected component may be classified as forward arcs, feedback arcs or reversing joins. There are three cases:
  (a) If the edge joins two in- or two out-sides then it must be a reversing join. Order and orientations remain unchanged.
  (b) If the edge joins one in- and one out-side and the out-side node precedes the in-side node, the edge is classified as forward arc and no reordering nor reorientation is needed.
  (c) If the edge joins one in- and one out-side and the in-side node precedes the out-side node, the algorithm checks whether there exists a forward arc path from the in-side node to the out-side node. The existence of the path forces the in-side node to precede the out-side node, so the edge is classified as a feedback arc. Otherwise reordering allowing classifying the edge as forward arc is computed using an adapted Pearce-Kelly algorithm (see Figure 8).

#### Data structure and time complexity

For the purpose of our algorithm we designed a data structure that provides a dynamic representation of node orientations, connected components and orders within components. It is based on the classical disjoint-set data structure of Hopcroft and Ullman (1973), which provides operations:

• FIND-SET(x) – return the representative of the set containing *x*,
• UNION(x,y) – merge sets containing *x* and *y* into a new one.

Implementation using disjoint-set forest with union by rank and path compression yields for both operations amortized cost O(α(n)), where *n* is the total number of set elements and α is the inverse of the Ackermann function (Cormen et al., 2009).

Connected components are represented by the sets of nodes. The above operations support checking whether the added edge joins nodes from the same or different components and, in the second case, merging components. In order to represent orientations and order we have to augment the disjoint-set structure. Current orientation of a node is encoded as either 1 (same as initial) or −1 (opposite, i.e. implying swapping in- and out-side and reverse complementing the labeling sequence). In order to encode the order within the component, nodes are bijectively assigned integers from some interval {i,i+1,…,j}, called *positions*.

Adding to each node attributes directly representing its orientation and position within the current component would waste the efficiency of UNION operation, because every node of one of merged components would require at least updating the position attribute. Therefore we introduced attributes that represent these features *locally*, i.e. with respect to nodes’ parents in the component tree. Namely, each node has the following attributes:

• *orient* – orientation relative to the parent, either 1 (the same) or −1 (opposite),
• *shift* – preliminary position relative to the parent and orientation, inherited by descendants,
• *reorder_shift* – final position relative to the preliminary position, non-inherited by descendants.

Actual orientation of a node is the product of *orient* attributes of this node and its ancestors in the tree. Actual position of the node is the sum of

• all *shift* attributes of node’s ancestors multiplied by their actual orientation and
• the sum of node’s *shift* and *reorder_shift* attributes multiplied by its actual orientation.

The attributes are updated during the path compression procedure of the disjoint-set structure. Since updating require constant time per node in the path, the asymptotic cost of the path compression is not affected. Moreover, every calculation of node’s orientation or position calls the path compression procedure, so its amortized cost is O(α(n)), where *n* is the number of nodes in the graph.

When a new edge is added to the graph, determining the respective case from subsection Adding edges to the graph requires calculating component representatives, orientations and positions of the ends of the edge. The subsequent steps modify the structure in the following way:

• In case 1 operation UNION is performed, followed by modifying attributes *shift* and (only in case 1.b.) *orient* in the root node of one component.
• In cases 2.a and 2.b the structure remains unchanged.
• In case 2.c attributes *reorder_shift* are modified in affected nodes when repositioning is required, otherwise the structure remains unaffected.

Consequently, in all cases except 2.c, the total amortized cost of adding the edge to the graph is O(α(n)). Processing case 2.c consists of the following steps:

• Identifying the set *V*_*F*_ of nodes from the affected region that are reachable from the in-side node (i.e. the set of green nodes in Figure 8). To this aim, an adapted depth-first search algorithm is called, which uses only forward arcs that don’t exit the affected region. Therefore, for each edge outgoing from each visited node, the algorithm needs to calculate the position of the target node. Consequently, the amortized cost of this step is $O\left(\left|E_{F}\right|\cdot \alpha \left(n\right)\right)$, where $E_{F}$ denotes the set of edges outgoing *V*_*F*_ nodes.
• Identifying the set *V*_*B*_ of nodes from the affected region, from which the out-side node is reachable (i.e. the set of blue nodes in Figure 8). Similarly, the amortized cost of this step is $O\left(\left|E_{B}\right|\cdot \alpha \left(n\right)\right)$, where $E_{B}$ denotes the set of edges leading to *V*_*B*_ nodes.
• Updating positions of the nodes in *V*_*F*_∪*V*_*B*_. This step is dominated by sorting *V*_*F*_ and *V*_*B*_ nodes according to their original positions, which has cost $O\left(\left|V_{F}\right|\text{log}\left|V_{F}\right|+\left|V_{B}\right|\text{log}\left|V_{B}\right|\right)$.

Therefore, the total cost of processing new edge in this case is $O\left(\left(\left|E_{F}\right|+\left|E_{B}\right|\right)\cdot \alpha \left(n\right)+\left|V_{F}\right|\text{log}\left|V_{F}\right|+\left|V_{B}\right|\text{log}\left|V_{B}\right|\right)$ and substantially depends on the size of the affected region and graph structure.

## Supplemental information

Table S1. GenBank accession numbers of *Escherichia coli* genomes used in real data experiments, related to Figures 4 and 5
